# Dataset generated in a systematic review and meta-analysis of biological clocks as age estimation markers in animal ecology

**DOI:** 10.1016/j.dib.2023.109615

**Published:** 2023-09-25

**Authors:** Louis-Stéphane Le Clercq, J. Paul Grobler, Antoinette Kotzé, Desiré Lee Dalton

**Affiliations:** a South African National Biodiversity Institute, Pretoria 0001, South Africa; b Department of Genetics, University of the Free State, Bloemfontein 9300, South Africa; c School of Health and Life Sciences, Teesside University, Middlesbrough TS1 3BA, United Kingdom

**Keywords:** Animals, Biological clocks, Epigenetics, Methylation, Telomeres, Biomarker, Age determination, Meta-analysis

## Abstract

The dataset comprises a comprehensive systematic review and meta-analysis exploring the utility of biological clocks as age estimation markers in the context of animal ecology. The systematic review adhered to PRISMA guidelines and employed optimized Boolean search strings to retrieve relevant studies from Scopus and Dimensions databases. A total of 78 methylation studies and 108 telomere studies were included after rigorous screening. Effect sizes were computed, and statistical transformations were applied when necessary, ensuring compatibility for meta-analysis. Data from these studies were meticulously collected, encompassing statistical measures, study attributes, and additional biological information. The dataset comprises several folders, carefully organized to facilitate access and understanding. It contains raw and processed data used in the systematic review and meta-analysis, including Boolean search strings, database search results, citation network analysis data, PRISMA statements, extracted study data, and input data for meta-analysis. Each folder's contents are described in detail, ensuring clarity and reusability. This dataset aggregates primary research studies spanning diverse ecosystems and taxa, providing a valuable resource for researchers, biodiversity managers and policymakers. This dataset offers a wealth of information and analysis potential for researchers studying age estimation markers in animal ecology, serving as a robust foundation for future investigations and reviews in this evolving field.

Specifications Table

**Table:** 

Subject	Biological Science (Aging, Genetics: Epigenetics)
Specific subject area	This dataset pertains to studies and study attributes for two biological clocks, methylation and telomeres, used in animal age determination.
Data format	Raw, Filtered, Analysed
Type of data	Tables, Figures
Data collection	A Boolean search string was used to search two scientific databases. Initial results were narrowed down by automated filtering techniques such as additional search terms as inclusion and exclusion criteria. Results were screened manually by assessing the titles, abstracts, and key words for relevance. Additional studies were identified from ancillary “free term” searches. The final set of preliminary studies for inclusion were sought for full text retrieval. Relationships between included studies were explored by citation network analysis of bibliographic coupling. Data collated from studies included species, sample size, and statistical measures used to calculate effect sizes and variances.
Data source location	Data included in the meta-analysis and review were collated from scientific literature (Scopus and Dimensions databases) and represents a globally distributed dataset from 2001 to 2023, with most studies originating from North America, Europe, and Australia. A smaller number of studies included sampling from South America and Africa. Institution: University of the Free State Address: 205 Nelson Mandela Dr, Park West, Bloemfontein, 9301 Co-ordinates: -29.1068, 26.1922
Data accessibility	Repository name: Zenodo Data identification number: 7091053 Direct URL to data: https://doi.org/10.5281/zenodo.7091053
Related research article	Le Clercq, L.-S., Kotzé, A., Grobler, J.P., and Dalton, D.L. (2023), Biological clocks as age estimation markers in animals: a systematic review and meta-analysis. *Biological Reviews*. https://doi.org/10.1111/brv.12992[1]

## 1 Value of the Data

• These datasets represent the most up-to-date collection of studies using two biological clocks based on epigenetics, methylation and telomere length, to determine age in animals and defines the state-of-the-art of the field using a systematic method that enables both transparency and reproducibility.
• This data helps gain novel insights into key attributes of study design such as method used, sample sizes, and tissue type and may guide the design of future studies.
• The side-by-side comparison of the overall utility of either method for age estimation helps provide clarity on the effectiveness of either method or may assist scientists when choosing a method of studying ageing in natural populations.
• While telomere length has been in use for two decades, methylation as a biomarker for age is a relatively new and rapidly evolving field, which may likely require an updated review on the topic in a few years; in which case this dataset serves as a good baseline for subsequent reviews.
• Authorship of included studies for both methylation and telomeres was used to create and benchmark a new measure of bias for meta-analysis, Author Bias, calculated by a custom PYTHON script called ABCal to quantitate the effects of highly represented authors.
• Study attributes, such as year of publication and study location, were also used to benchmark scientometric plotting functionality using the same tool.

## 2 Data Description

The full dataset consists of several folders and subfolders containing the raw and/or processed data used in the systematic review and meta-analysis. The first folder is named “literature search” and contains three subfolders. The first contains two text files with the Boolean search strings, used to search databases, while the second and third contains the raw results from the search of the Scopus and Dimensions databases as comma separated value files. The column headings for search result files are described in Tables 1 and 2. The second folder is named “Citation Network Analysis” and contains two subfolders, one for raw data and one for results. The data folder contains two files for the methylation studies and two for the telomere studies, of which the comma separated value file contains the studies selected for inclusion after screening, in the same format as Scopus output, while the text file contains the same data transformed to match the input requirements for citation network analysis. The column headings for the transformed data are described in Table 3. The results subfolder contains the images for the citation network analysis. The third folder named “PRISMA Statements” contains the two PRISMA statements for methylation and telomere studies respectively. The fourth folder is named “Extracted Data” and contains two subfolders. The first subfolder is titled “Extracted Data – Studies”, and contains a spreadsheet workbook with three tabs, one for methylation studies and two for telomere studies; column headings and associated data is described in Table 4. The second subfolder is titled “Extracted Data – Other” and contains data that was extracted from individual studies where relevant statistics could not be retrieved from the full text. The fifth folder is titled “Meta-Analysis” and contains that final dataset in comma separated value format that was used to perform the meta-analysis. The column headings and associated data is described in Table 5. The same folder also contains the related forest plots that were generated as output. The sixth and final folder is named “Code” and contains the R code used to perform the meta-analysis. This file is in the standard R format (.R) and contains relevant labels and annotations to explain which steps were performed by specific lines of code.

**Table 1:** Field names and data description for search results retrieved from Scopus.

Field name	Data
Authors	Authors listed by surname and first initials
Author(s) ID	Authors listed by Scopus ID
Title	Main title of the article
Year	Year of publication
Source title	Name of journal in which article is published
Volume	Volume number for article in journal (if available)
Issue	Issue number for article in journal (if available)
Art. No.	Article number in journal, typically used for online only articles
Page start	Page in volume/issue where article starts
Page end	Page in volume/issue where article ends
Page count	Total number of pages
Cited by	Total number of citations
DOI	Digital Object Identifier (DOI) for article
Link	URL to online page for article at publisher
References	Full list of references cited in the article
Document Type	Type of publication e.g., article, review, book etc.
Publication Stage	Status of publication e.g., final or in press
Open Access	Open access status e.g., green or gold
Source	Database used as source to retrieve document details e.g., Scopus
EID	Electronic Identifier (EID), usually the last part of DOI

**Table 2:** Field names and data description for search results retrieved from dimensions.

Field name	Data
Publication ID	Unique identifier for publication of Dimensions database
DOI	Digital Object Identifier (DOI) for publication
Title	Main title of the publication
Abstract	Abstract for publication
Source title/Anthology title	Name of journal/source of publication
PubYear	Year of publication
Volume	Volume number for publication in journal (if available)
Issue	Issue number for publication in journal (if available)
Pagination	Start and end page numbers for publication
Authors	Authors listed by surname and name or initials
Authors Affiliations - Name of Research organization	List of affiliations (organization) of authors
Authors Affiliations - Country of Research organization	List of affiliations (country) of authors
Dimensions URL	URL to online page for publication on Dimensions
Times cited	Total number of citations
Cited references	Full list of references cited in publication

**Table 3:** Field names and data description for Scopus results after transformation for citation network analysis.

Field name	Data
AU	Authors listed by surname and initials
TI	Title of publication
PY	Publication year
SO	Name of journal for publication
VL	Volume number for publication in journal
BP	Start/beginning page for publication in volume
DI	Digital Object Identifier (DOI) for publication
CR	List of all cited references

**Table 4:** Field names and data description for file containing extracted study data from the review.

Field name	Data
Generic name	Common name (English) for species
Scientific name	Latin binomial for species
Author	Study label as first author and publication year e.g., Author et al, 2020
Sample size	Total sample size
R-squared	Correlation coefficient for relationship
p-value	Significance level as per reported probability value
F	Value for F-test statistic (if applicable)
X	Value for Chi-squared test statistic (if applicable)
t	Value for t-test statistic (if applicable)
Outcomes	Tissue type used to measure values
Fishers-Zr	Computed effect size expressed as Fisher's-Z
Var	Computed variance of the effect size

**Table 5:** Field names and data description for file containing extracted data prepared for meta-analysis.

Field names	Data
Generic name	Common name (English) for species
Scientific name	Latin binomial for species
Class	(1) Vertebrate class e.g., Fish, Amphibian, Reptile, Bird, Mammal (All)
	(2) Grouping of birds e.g., Chats & Flycatchers (Birds)
Class.Num	Numeric encoding for class from 1 to 5
Mammal.Group	Mammal group per systematic review e.g., Aquatic, Carnivore, Primate etc.
Author	Study label as first author and publication year e.g., Author et al, 2020
Group	Grouping based on model type per the review*
N	Sample size used in study
Cor	Correlation coefficient used directly in methylation meta-analysis
Genome.Size	Size of the full genome given in billions of base pairs (methylation)
Karyotype	Total number of chromosomes (telomeres)
Tissue	Tissue type used to measure attributes
Year	Publication year
Method	Quantitative method used to make measurements e.g., PCR
Life Min	Lower end of range for life expectancy
Life Med	Middle value of range for life expectancy
Life Max	Upper end of range for life expectancy
Bias	Level of potential bias from authorship as Low, Medium, or High
Cal.Bias	Raw value for the normalised calculated author bias values

⁎ Group 1: single species for model, validated in same species; Group 2: single species for model, validated in different/related species; Group 3: multiple species for model, validated in multiple species.

## 3 Experimental Design, Materials and Methods

### 3.1 Literature search and study screening for systematic review

**Fig. 1:**
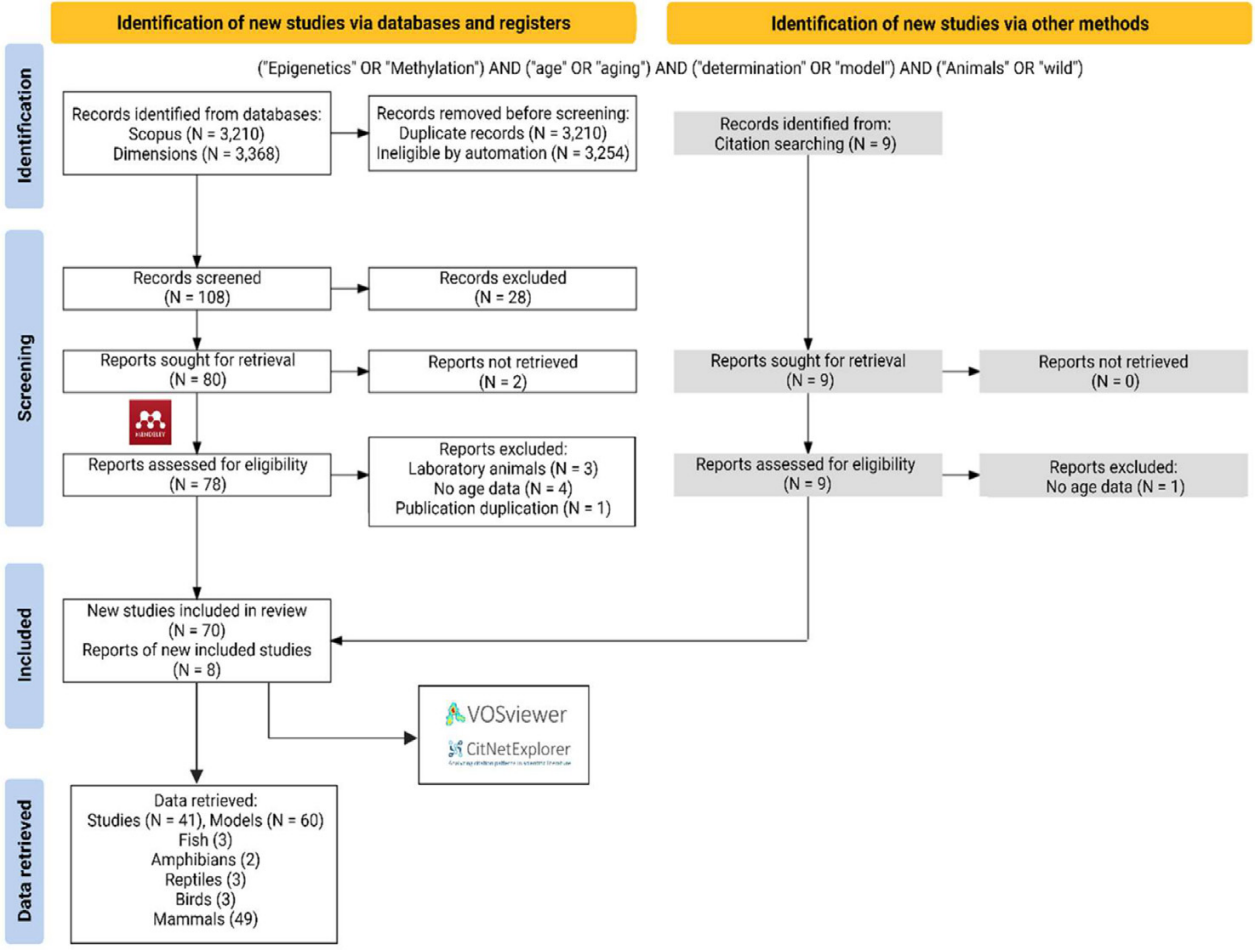
PRISMA statement for the systematic approach used to identify studies that measured methylation in relation to age to develop methylation as a biomarker for age in animals. Two databases were searched using the indicated Boolean search strings. Initial automated screening removed duplicates and used additional key words to filter the results. Potential studies from the cleaned dataset were sought for retrieval and assessed for eligibility in Mendeley. Additional studies were identified from citation searches. Details are provided for relevant exclusion criteria used at each step. The final set of included studies were analysed by citation network analyses to facilitate the synthesis of the literature. Further details are also provided for the retrieval of model details and summary statistics from individual studies for inclusion in meta-analysis. (image edited in BioRender.com).

**Fig. 2:**
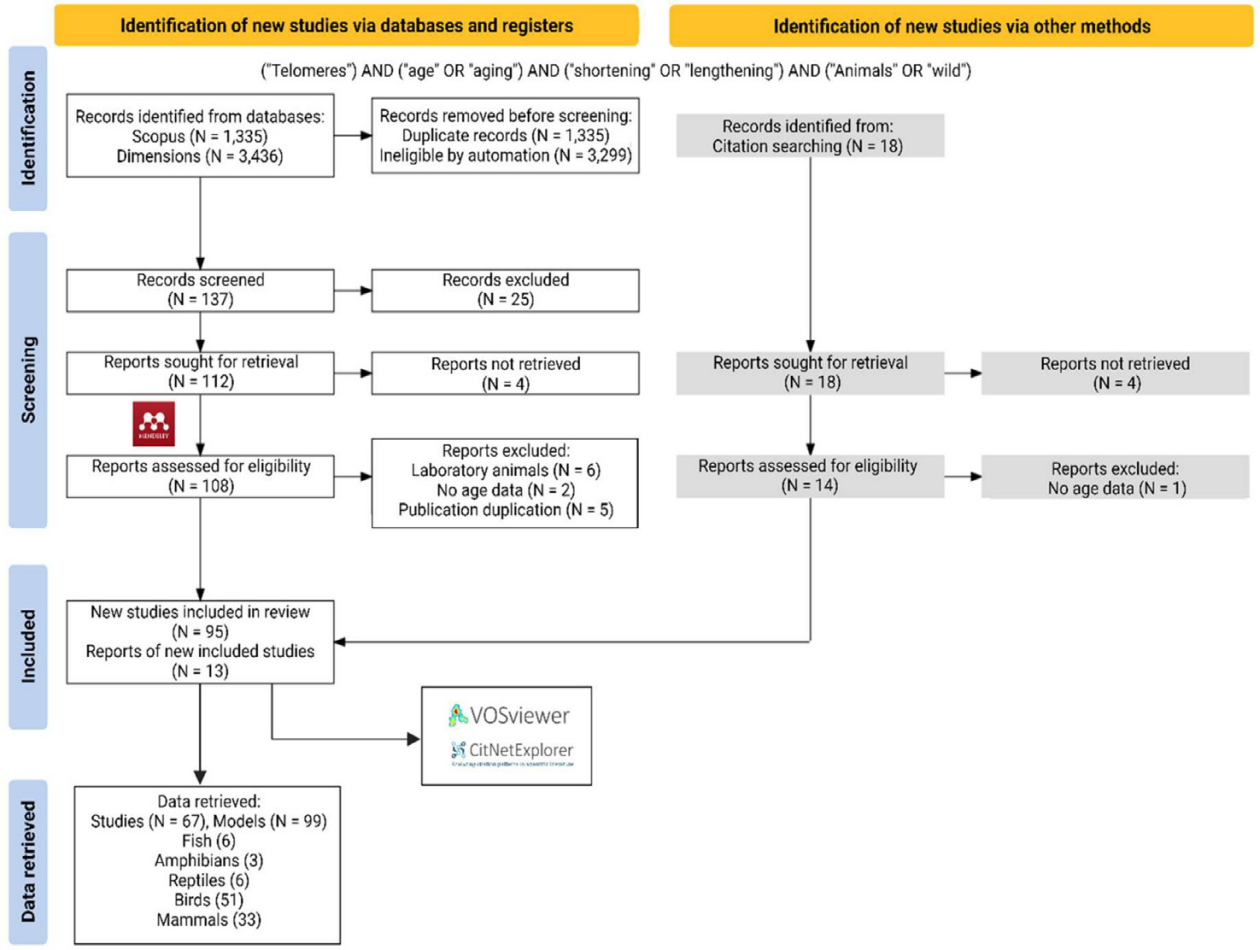
PRISMA statement for the systematic approach used to identify studies that measured changes in telomere length in relation to age to develop telomere length as a biomarker for age in animals. Two databases were searched using the indicated Boolean search strings. Initial automated screening removed duplicates and used additional key words to filter the results. Potential studies from the cleaned dataset were sought for retrieval and assessed for eligibility in Mendeley. Additional studies were identified from citation searches. Details are provided for relevant exclusion criteria used at each step. The final set of included studies were analysed by citation network analyses to facilitate the synthesis of the literature. Further details are also provided for the retrieval of model details and summary statistics from individual studies for inclusion in meta-analysis. (Image edited in BioRender.com).

**Fig. 3:**
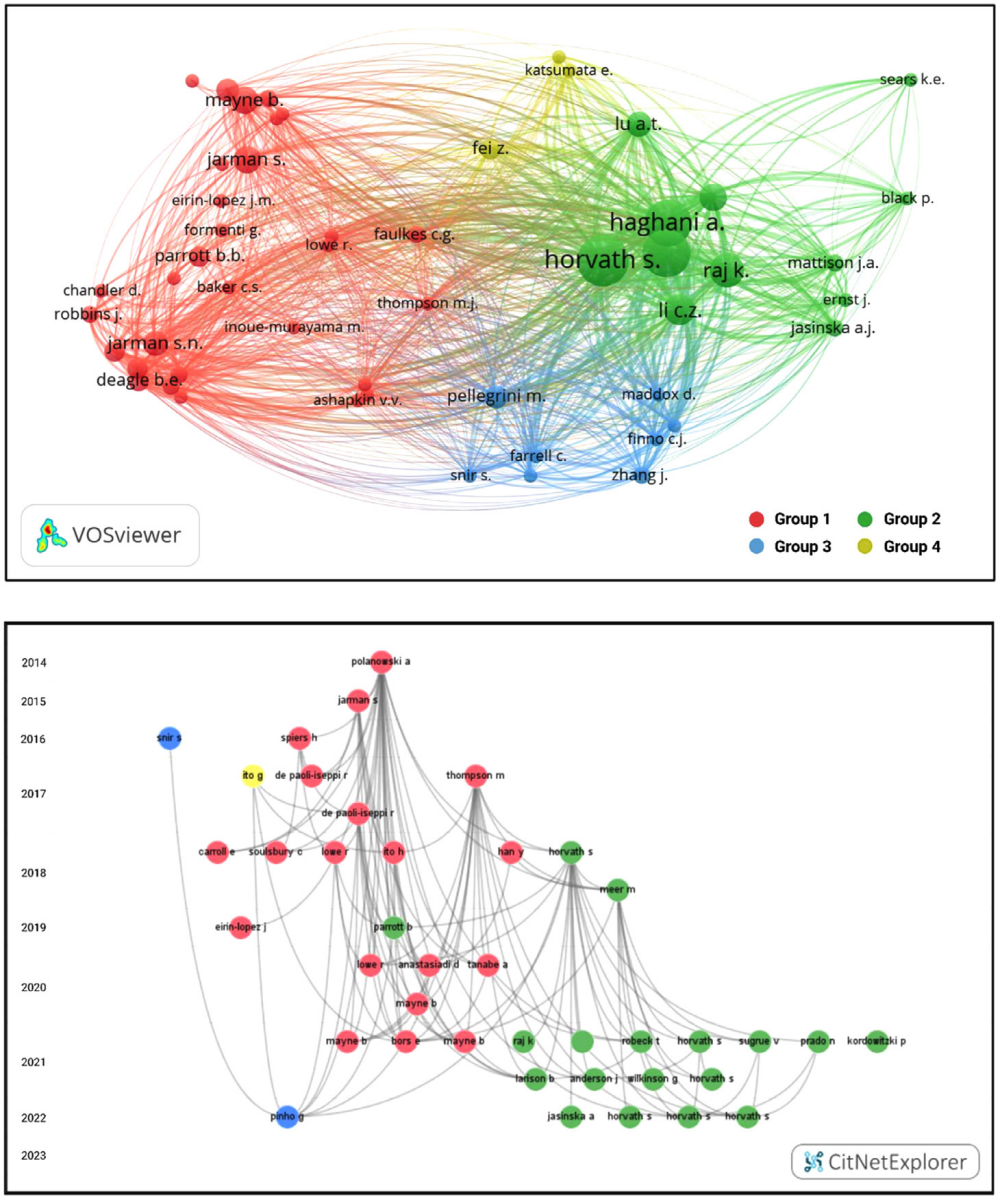
Visualised citation network for methylation studies identified in database literature searches, visualised in VOSviewer in CitNetExplorer. The top panel indicates clustering analyses performed in VOSviewer, which identified four key groups, labelled 1 through 4. The bubbles indicate key authors labelled by surnames and initials with bubble size corresponding to the number of citation links with other authors. The bottom panel indicates citation network analyses of publications in CitNetExplorer, which are organized by year (2014–2023) with the name and first initial of the first author indicating individual studies. The relationship between studies by virtue of co-citations in the reference lists are indicated by grey lines. Subgroup analyses identified four key clusters, indicated according to the group colours from VOSviewer. (Image edited in BioRender.com).

**Fig. 4:**
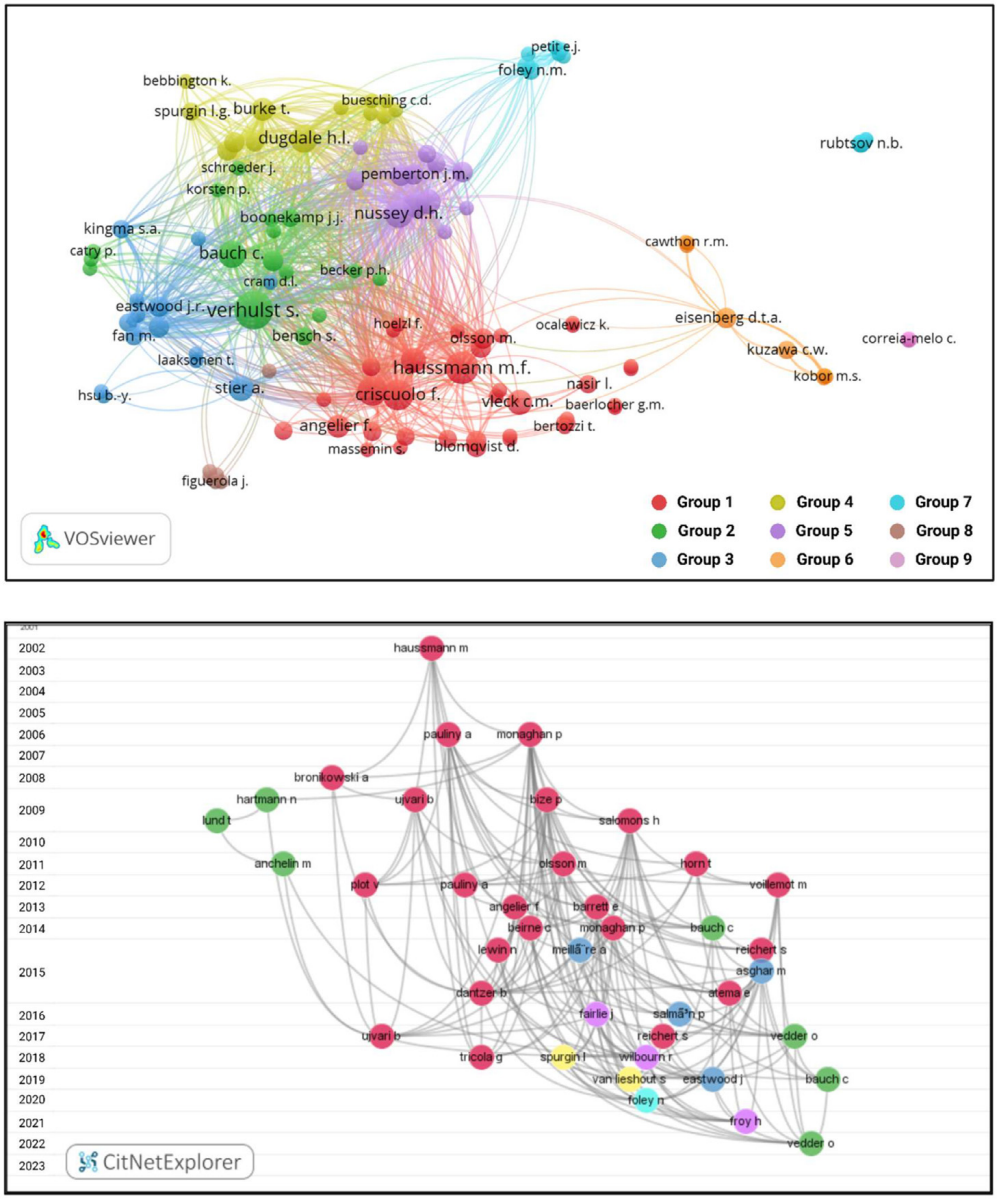
Visualised citation network for telomere studies identified in database literature searches, visualised in VOSviewer in CitNetExplorer. The top panel indicates clustering analyses performed in VOSviewer, which identified nine key groups, labelled 1 through 9. The bubbles indicate key authors labelled by surnames and initials with bubble size corresponding to the number of citation links with other authors. The bottom panel indicates citation network analyses of publications in CitNetExplorer, which are organized by year (2002–2023) with the name and first initial of the first author indicating individual studies. The relationship between studies by virtue of co-citations in the reference lists are indicated by grey lines. Subgroup analyses identified several key clusters, indicated according to the group colours from VOSviewer. (Image edited in BioRender.com).

Literature was searched and screened using systematic review methods (Figs. 1 and 2) per the preferred reporting items for systematic reviews and meta-analysis (PRISMA) statement [2,3], in line with PRISMA Ecology and Evolution guidelines [4] and Cochrane best practices [5]. Literature was searched between September of 2022 and June of 2023 on two databases: Scopus (www.scopus.com) and Dimensions (www.dimensions.ai). Databases were searched using an optimized Boolean search string derived from the PICO terms for the aim and objectives of the review. For methylation studies the search string was: (“Epigenetics” OR “Methylation”) AND (“age” OR “aging”) AND (“determination” OR “model”) AND (“Animals” OR “wild”). For telomere studies the search string was: (“Telomeres”) AND (“age” OR “aging”) AND (“shortening” OR “lengthening”) AND (“Animals” OR “wild”). Initial results were subjected to further automated screening using additional search terms as constraints to reduce the results for specificity and to exclude results from human studies. For the Scopus and Dimensions database searches, the final set of results for screening were exported in the comma separated value (CSV) format. Sources identified were imported (citation and abstract) into Mendeley citation manager (www.mendeley.com) for manual screening. The final set of studies that passed preliminary screening were sought during full text retrieval and added to the imported references if it wasn't already included. A total of 78 studies were included in the final review for methylation and 108 for telomeres. Further analyses of included studies were done through citation network analyses. For the Scopus database, the results were merged and reformatted with the R package *Scopus2CitNet 0.1.0.0* in RStudio 1.4.1106 [6], running R 4.0.5 [7]. The final included studies from the results of Scopus and Dimensions were subsequently visualized by year in CitNetExplorer 1.0.0. and by group in VOSviewer 1.6.16 [8] keeping only those papers that overlapped in terms of references cited (bibliometric coupling) for the largest connected components (Figs. 3 and 4).

### 3.2 Data collection and processing for meta-analysis

Data was collected from studies that reported models using methylation or telomeres as biomarker to infer the age of animals. Relevant statistics and study attributes were retrieved from tables, figures, or the main text. For methylation studies this included key reported statistics such as the *correlation coefficient* and *p-values* for models. For telomere studies, several different statistical tests were applied beyond linear models. As such, the reported statistics compiled included measures from tests reported as *correlation coefficients* and *p-values* as well as *t-values, F-values*, and *z-values*. Where clear statistical measures were not available, WebPlotDigitizer 4.6 [9] was used to extract data from graphs or plots to repeat the reported tests and derive the relevant statistics. Data was extracted from figures from one methylation study, for snow leopards [10], and two telomere studies, for baboons [11] and chimpanzees [12], for which the correlation coefficients were computed using linear regression. For telomere studies, data was also retrieved from the online supplements to compute relevant statistics for rainbow trout [13] and stonechat species [14], for which the F-statistics were compute using ANOVA. Key study characteristics such as species, sample size, tissue type, and empirical method were also collected. Additional attributes such as lifespan, karyotype, and genome size were retrieved from online databases [15], [16], [17], [18].

The effect sizes of the treatment effect (TE), expressed as *Fisher's-Z*, as well as the variance thereof expressed as standard error (SETE) were computed with the R package *compute effect size 0.2-2* [19] based on equations derived from “The Handbook of Research Synthesis and Meta-Analysis” [20]. Where the *correlation (r)* was available the z-transformed effect sizes, *Fisher's-Z*, were calculated as per equation 1.

(1) $$Fisher{}^{\prime }s\;Z=0.5\;\times \log\left(1+r\right)\;/\;\left(1-r\right)$$

where the *correlation (r)* was not available, the *Fisher's-Z* was derived from reported statistical measures by first converting between the given measure and the correlation. For *Chi-squared (χ^2^)*, equation 2 was used, where the correlation is derived from the quotient of the square root of *Chi-squared* and *sample size (n)*.

(2) $$r=\;\sqrt{\chi 2/n}$$

For F-test statistics *(f)*, equation 3 was used to first calculate *Cohen's d (d)* by taking the square root of the *f-value* multiplied by the sum of group sizes divided by the product of group sizes. From *d*, the correlation was derived using equation 4, where *d* is divided by the square root of the sum of (i) the square of *d* and (ii) the quotient of the sum of squared group sizes and the product of group sizes.

(3) $$d=\;\sqrt{f\times \;\left(n_{1}+n_{2}\right)\;/\;\left(n_{1}\times n_{2}\right)}$$

(4) $$r=d\;/\;\sqrt{d^{2}+{\left(n_{1}+n_{2}\right)}^{2}\;/\;\left(n_{1}\times n_{2}\right)}$$

For t-test *(t)* statistics, equation 5 was used to calculate the correlation by taking the square root of the quotient between (i) the square of the *t-value* and (ii) the sum of *t-squared* added to *sample size (n)*, subtracting two.

(5) $$r=\sqrt{t^{2}\;/\;\left(t^{2}+n-2\right)}$$

where only the *probability (p-value)* was available, the correlation was derived from first calculating *Cohen's d* using the quantile function of the Student's t-distribution (where *q* represents the quantile function, *α* represent the desired significance level from the *p-value*, and *df* represents the degrees of freedom from the sample size) and multiplying by the square root of the quotient for the sum of group sizes and the product of group sizes (equation 6). Hereafter the correlation could be calculated as per equation 4.

(6) $$d=q_{df}^{\alpha }\times \sqrt{\left(n_{1}+n_{2}\right)\;/\;\left(n_{1}\times n_{2}\right)}$$

The variance associated with individual correlations, *var_r_*, was calculated using equation 7 by dividing (i) the square of *r-squared* subtracted from one by (ii) the *sample size (n)* minus one.

(7) $$var_{r}={\left(1-r^{2}\right)}^{2}\;/\;\left(n-1\right)$$

This data was incorporated into a systematic review and meta-analysis as previously published [1].

## 4 Limitations

Tests for funnel plot asymmetry as a measure of potential publication bias were statistically significant (p-value < 0.05), indicating the potential absence of studies from the primary literature. This is likely due to small study effects, where studies with smaller sample sizes are likely excluded from publication through peer-review. The low levels of studies reported from the global South and Africa may also indicate that similar studies from these regions are missing from the primary literature, possibly due to a lack of priority or suitable resources. The trim-and-fill method was used to infer possibly missing studies and, while this did not substantially alter the overall interpretations of the results, 18 studies were inferred and added as missing from the methylation dataset while 49 studies were inferred and added as missing from the telomere studies. Additionally, it should be noted that the methylation dataset contained an abundance of studies in mammals, with fewer studies from other vertebrate classes, while the telomere studies included an abundance of studies in birds. There was, however, little evidence that vertebrate class had a significant effect on the measured attributes. Lastly, differences in timespan for publications may impact interpretations as reported effect sizes are known to show temporal variation; typically studies that show large effect sizes are published first, establishing the validity of a method or introducing the field, however, later studies—with smaller effect sizes—are often published a decade later. Given that methylation studies have only been published over half of the same period than for telomere studies, future studies may still be published for methylation showing lower effect sizes.

## 5 Ethics Statement

Data used in our review represent secondary data from published literature and online resources. Included studies complied with the ARRIVE guidelines and were carried out in accordance with the United Kingdom (UK) Animals (Scientific Procedures) Act, 1986 and associated guidelines; European Union Directive 2010/63/EU for animal experiments; the National Institute of Health (UK) guide for the care and use of laboratory animals (NIH Publications No. 8023, revised 1978); or other ethical guidelines as per the country of origin. Ethics approvals for the present study were obtained from the University of the Free State (approval number: UFS-AED2020/0015/1709) as well as the South African National Biodiversity Institute (approval number: SANBI/RES/P2020/30).

## CRediT authorship contribution statement

**Louis-Stéphane Le Clercq:** Conceptualization, Methodology, Data curation, Formal analysis, Writing – original draft, Writing – review & editing, Funding acquisition. **J. Paul Grobler:** Writing – review & editing, Resources, Supervision. **Antoinette Kotzé:** Writing – review & editing, Resources, Supervision. **Desiré Lee Dalton:** Conceptualization, Writing – review & editing, Resources, Supervision.

## Data Availability

Biological clocks (Data) (Reference data) (Zenodo).
